# SNFing HIV transcription

**DOI:** 10.1186/1742-4690-3-49

**Published:** 2006-08-09

**Authors:** Michael Bukrinsky

**Affiliations:** 1 The George Washington University, Department of Microbiology, Immunology and Tropical Medicine, Washington, DC 20037, USA

## Abstract

The SWI/SNF chromatin remodeling complex is an essential regulator of transcription of cellular genes. HIV-1 infection induces exit of a core component of SWI/SNF, Ini1, into the cytoplasm and its association with the viral pre-integration complex. Several recent papers published in EMBO Journal, Journal of Biological Chemistry, and Retrovirology provide new information regarding possible functions of Ini1 and SWI/SNF in HIV life cycle. It appears that Ini1 has an inhibitory effect on pre-integration steps of HIV replication, but also contributes to stimulation of Tat-mediated transcription. This stimulation involves displacement of the nucleosome positioned at the HIV promoter.

Transcription of integrated HIV genome, as well as other genes, has to deal with nucleosomes that cover DNA and limit its access to transcription machinery. Interestingly, regardless of the integration site, nucleosomes are found at specific positions within the HIV LTR [1]. Transcription is initiated within the nucleosome-free region between nuc-0 and nuc-1, and leads to remodeling of nuc-1, which is positioned immediately downstream of the transcription start site. The remodeling appears to be specific for nuc-1 [1], suggesting involvement of Tat in this process. However, the mechanisms responsible for this remodeling and possible activating effect on transcription remained elusive.

Integrase-interacting protein 1 (Ini1) was identified as an HIV IN-interacting factor in a two-hybrid screening [2]. The fact that Ini1, also known as SNF5, is the core sub-unit of the ATP-dependent chromatin remodeling complex SWI/SNF, which regulates expression of numerous eukaryotic genes by altering DNA-histone interactions [3-5], raised the possibility that Ini1 may contribute to preferential selection of transcriptionally active genes as integration sites of HIV-1 [6]. This notion was consistent with the finding that Ini1 stimulated IN activity *in vitro *[2] and was recruited from the nucleus to incoming pre-integration complexes (PICs) of HIV-1 [7]. One proposed model postulated that SNF5/Ini1 could target PICs to regions of the genome that are enriched for the SWI/SNF complex [8].

Two new reports published in this issue of Retrovirology, as well as several papers in other journals [9-11], challenge the older findings and suggest a new role for SWI/SNF in HIV replication. The study by Trono and colleagues [12] was designed to investigate the potential role of Ini1 in HIV-1 integration. Inactivation of Ini1 using RNA interference did not reduce the transduction efficiency of the VSV-G-pseudotyped HIV-1-based vector system, arguing against Ini1 involvement in HIV integration. This conclusion was supported by effective transduction of Ini1-deficient cells derived from a malignant rhabdoid tumor by an HIV-based vector, a result corroborating previous findings [13]. Using a similar approach, Emiliani and colleagues [9] observed that silencing of SNF5/Ini1 resulted in an increase in the formation of 2-LTR circle and integrated forms of HIV-1 DNA, leading to increased transduction efficiency and HIV-1 replication. This result suggests that SNF5/Ini1 may participate in an anti-viral cellular response. The reason Ariumi and co-authors [12] did not observe increased transduction efficiency in cells where Ini1 had been silenced may be due to the use of VSV-G-pseudotyped constructs in their experiments. Such constructs enter target cells via endocytosis rather than fusion with the plasma membrane, thus limiting exposure of their PICs to cytosolic Ini1.

Surprisingly, Ariumi et al. also reported that HIV-1 replication in Ini1 knockdown cells was significantly reduced [12]. They found that Ini1 synergizes with Tat to stimulate transcription from HIV LTR. Moreover, Ini1 could be co-immunoprecipitated with Tat, and both Rpt1 and Rpt2, two direct imperfect repeats required for the formation of a functional SWI/SNF complex, participated in co-activation of Tat-mediated HIV-1 transcription. The activation of Tat-mediated transcription by Ini1, although not observed by Maroun et al. [9], corroborates results of Mahmoudi et al. who reported that Ini1, p300 acetyltransferase and Tat cooperate to activate the HIV promoter [11]. Acetyltransferase activity of p300 was crucial for this cooperative effect, as well as the Tat lysines 50 and 51, which are the targets of acetylation by p300 [14-16]. Interestingly, Tat acetylation on lysines 50 and 51 was found necessary for interaction with BRG1 [11], a catalytic subunit of SWI/SNF complexes (unfortunately, the role of Tat acetylation in its interaction with Ini1 has not been reported). On the other hand, acetylation on lysine 50 prevented interaction of Tat with BRM [10], another DNA-dependent ATPase of SWI/SNF complexes closely related to BRG1 [17]. Therefore, it appears that Tat can recruit different SWI/SNF complex subunits to the HIV-1 promoter in an acetylation-regulated fashion, resulting in a dramatic amplification of the Tat activity.

How does the SWI/SNF complex stimulate Tat activity? A hint to the possible mechanism of this effect was provided by Agbottah et al. [18]. Consistent with results reported by Mahmoudi et al. [11], they found that Tat acetylated on lysines 50 and 51 interacts with BRG1 and both associate with the activated HIV-1 promoter. Using chromatin immunoprecipitation assay, Agbottah and co-authors demonstrated that both Tat and BRG1 can localize to the site that, in an unactivated HIV promoter, is occupied by nuc-1 [1]. Consistently, the nuc-1-binding site on HIV LTR became highly susceptible to digestion by restriction enzyme Afl II when BRG1 and acetylated Tat were added to the in vitro transcription reaction performed with chromatinized HIV promoter. Neither Tat not BRG1 alone produced this effect, suggesting that BRG1 may remodel nuc-1 in a Tat-dependent fashion.

Therefore, the following model can be proposed (Fig. 1). Following initiation of transcription from the HIV promoter, Tat binds to newly synthesized TAR RNA and recruits several factors, including acetyltransferase p300 and, via interaction with Ini1 and BRM, the SWI/SNF complex. The latter initiates remodeling of nuc-1. In the meantime, acetylation of Tat on lysine 50 by p300 leads to dissociation of Tat from TAR and creates a binding site for another acetyltransferase, pCAF. Acetylated Tat recruits another SWI/SNF complex, this time via Ini1 and BRG1 subunits, which complete remodeling of nuc-1 and allow elongation of transcription.

**Figure 1:**
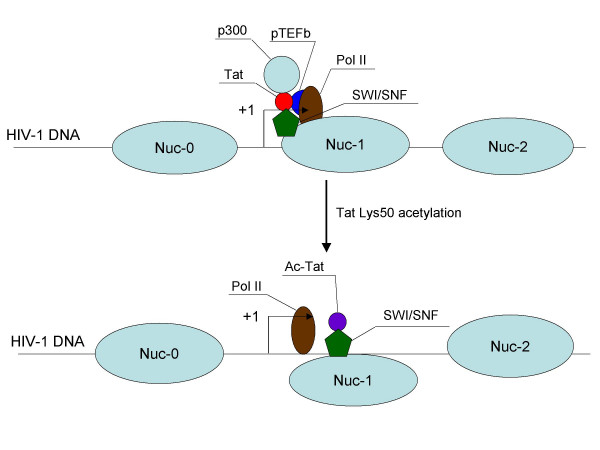
**A model depicting mechanisms of Nuc-1 remodeling during HIV-1 transcription**. +1 denotes the transcription start site in the HIV-1 LTR. Basal HIV promoter shows an elongation defect due to deficient loading of the transcriptional elongation complex pTEFb. Tat binding to the TAR stem-loop in the nascent viral RNA recruits pTEFb, which phosphorylates the C-terminal domain of RNA pol II and increases transcriptional elongation. Via interaction with the BRM, a catalytic subunit of SWI/SNF complexes, and a core subunit Ini1/SNF5, Tat also recruits the SWI/SNF complex, which initiates remodeling of nuc-1. Subsequent acetylation of the Tat lysine 50 by p300 results in Tat dissociation from TAR, but creates the binding sites for another SWI/SNF catalytic subunit, BRG1. The SWI/SNF complex recruited by the Tat acetylated on lysine 50 (which may be different from the one recruited by the TAR-bound Tat) completes remodeling of nuc-1 and allows the efficient elongation of transcription. See text for details.

Several issues remain to be resolved. Both BRM and BRG1 can associate with Tat, and acetylation on lysine 50 was found to promote Tat binding to BRG1 but inhibit binding to BRM. Tat transcriptional activity is regulated by acetylation, with acetylation at Lys 28 by pCAF promoting Tat association with pTEFb complex, which phosphorylates the C-terminal domain of RNA polymerase II and stimulates elongation of transcription, and acetylation at Lys 50 by p300 leading to the dissociation of Tat from TAR RNA [15]. Therefore, TAR-bound Tat may recruit BRM-containing SWI/SNF, whereas BRG1-containing SWI/SNF complex may be recruited following Tat acetylation by p300. Do BRM- and BRG1-containing SWI/SNF complexes perform different functions at the HIV LTR? Two subclasses of SWI/SNF complexes have been described in eukaryotes: SWI/SNF-α/BAF associated with BRG1 or BRM, and SWI/SNF-β/pBAF associated with BRG1 only [17]. The activities of these two types of SWI/SNF complexes differ, so a better characterization of LTR-bound SWI/SNF complexes would be necessary to determine their role in HIV transcription. Another remaining question is whether chromatin remodeling by SWI/SNF requires active transcription or just transcription initiation. Agbottah et al. reported that α-amanitin, which specifically blocks Pol II-dependent transcription, eliminated the nuc-1 remodeling by Tat and BRG1 [18]. This finding is in direct conflict with previously reported results that remodeling of nuc-1 is insensitive to α-amanitin [1]. Another controversy awaiting its resolution concerns activities of Ini1, which appears to inhibit a pre-integration step of HIV replication [9] but stimulates transcription of the integrated provirus [11,18]. It will be important also to determine the role of pCAF in nuc-1 remodeling. Finally, the role of Ini1 associated with the HIV pre-integration complex remains unclear. Sniffing of SWI/SNF functions in HIV transcription has just begun and many exciting findings can be expected in the near future.
